# Gestational Diabetes Mellitus in Africa: A Systematic Review

**DOI:** 10.1371/journal.pone.0097871

**Published:** 2014-06-03

**Authors:** Shelley Macaulay, David B. Dunger, Shane A. Norris

**Affiliations:** 1 Medical Research Council/Wits Developmental Pathways for Health Research Unit, Department of Paediatrics, University of the Witwatersrand, Johannesburg, South Africa; 2 Department of Paediatrics, University of Cambridge, Cambridge, United Kingdom; University of Perugia, Italy

## Abstract

**Background:**

Gestational diabetes mellitus (GDM) is any degree of impaired glucose tolerance first recognised during pregnancy. Most women with GDM revert to normal glucose metabolism after delivery of their babies; however, they are at risk of developing type 2 diabetes later in life as are their offspring. Determining a country’s GDM prevalence can assist with policy guidelines regarding GDM screening and management, and can highlight areas requiring research. This systematic review assesses GDM prevalence in Africa.

**Methods and Findings:**

Three electronic databases were searched without language restrictions; PubMed, Scopus and the Cochrane Library. Thirty-one search terms were searched. Eligible articles defined GDM, stated what GDM screening approaches were employed and reported GDM prevalence. The reporting quality and risk of bias within each study was assessed. The PRISMA guidelines for systematic reviews were followed. The literature search identified 466 unique records. Sixty full text articles were reviewed of which 14 were included in the systematic review. One abstract, for which the full text article could not be obtained, was also included. Information regarding GDM classification, screening methods and prevalence was obtained for six African countries; Ethiopia (n = 1), Morocco (n = 1), Mozambique (n = 1), Nigeria (n = 6), South Africa (n = 4) and Tanzania (n = 1). Prevalence figures ranged from 0% (Tanzania) to 13.9% (Nigeria) with some studies focussing on women with GDM risk factors. Most studies utilised the two hour 75 g oral glucose tolerance test and applied the World Health Organization’s diagnostic criteria.

**Conclusions:**

Six countries, equating to 11% of the African continent, were represented in this systematic review. This indicates how little is known about GDM in Africa and highlights the need for further research. Considering the increasing public health burden of obesity and type 2 diabetes, it is essential that the extent of GDM is understood in Africa to allow for effective intervention programmes.

## Introduction

Diabetes mellitus (DM) is a group of conditions that contribute significantly to the increasing health and financial burden in many countries around the world [1]. The prevalence of and screening methods for the clinical subgroups, type 1 diabetes mellitus and type 2 diabetes mellitus, are relatively well researched and understood in most countries. However, those pertaining to the subgroup known as gestational diabetes mellitus (GDM) are less established [2]. Gestational diabetes mellitus is defined by the World Health Organization as being “any degree of glucose intolerance with onset or first recognition during pregnancy” and should therefore include glucose readings that fall within the impaired glucose tolerance (IGT) diagnostic range, as well as those within the diagnostic range for diabetes [3], [4]. More recently, the American Diabetes Association defines GDM as “diabetes diagnosed during pregnancy that is not clearly overt diabetes” [5].

Pregnancy itself induces changes in maternal glucose metabolism and insulin sensitivity. As pregnancy progresses the demand for insulin production on the mother’s pancreas increases. In most instances, pregnant women are able to meet the increased insulin demand but in some cases these needs are not met resulting in poor glycaemic control and consequently GDM. Certain factors including having a family history of diabetes, being over 25 years of age, being obese, belonging to a particular ethnic group (African American, Hispanic, Indian) and having previously given birth to a baby weighing 4 kg or more (macrosomia), put women at greater risk of developing GDM [6], [7].

Pregnancies affected by GDM pose a risk for adversities such as the need for Caesarean sections due to fetal macrosomia. Macrosomia occurs as a result of accelerated fetal growth fuelled by maternal hyperglycaemia [8]. In approximately 95% of GDM cases maternal glucose metabolism returns to normal after delivery of the baby [9], however, an association between GDM and the development of type 2 diabetes mellitus in the mother later in life exists [10], [11]. In addition, research into the long term effects of poor maternal glucose metabolism on the fetus has revealed that offspring born to mothers with GDM are susceptible to IGT and obesity [12], [13]. With these associations in mind it would be important to identify pregnant women at risk for GDM so that prevention management such as lifestyle modifications can be implemented [14].

Consensus regarding screening for and classification of GDM is yet to be achieved globally [2]. The most recognised diagnostic test for GDM is the oral glucose tolerance test (OGTT) usually performed between 24–28 weeks gestation [15]. Different screening regimes for GDM exist and as a result studies investigating prevalence of GDM are often diverse in terms of methods employed, cut-off values used and consequently, results obtained [16]. Table 1 summarises some of the different screening regimes and respective glucose cut-off values used to diagnose GDM.

**Table 1:** The different diagnostic criteria available for the diagnosis of gestational diabetes mellitus.

Group/Organisation	Screening test	Diagnostic criteria: blood glucose level thresholds
American Diabetes Association [5], [52]	One step: 2 hr 75 g OGTT	At least one of the following must be met:
		Fasting: ≥5.1 mmol/l (92 mg/dl)
		1 hr: ≥10.0 mmol/l (180 mg/dl)
		2 hr: ≥8.5 mmol/l (153 mg/dl)
	OR Two step:	OR
	1) 1 hr 50 g (non-fasting) screen	If 1 hr: ≥10.0 mmol/l (180 mg/dl) proceed with step 2
	2) 3 hr 100 g OGTT	3 hr: ≥7.8 mmol/l (140 mg/dl)
Carpenter and Coustan [53]	3 hr 100 g OGTT	At least two of the following must be met:
		Fasting: ≥5.3 mmol/l (95.4 mg/dl)
		1 hr: ≥10.0 mmol/l (180 mg/dl)
		2 hr: ≥8.6 mmol/l (154.8 mg/dl)
		3 hr: ≥7.8 mmol/l (140 mg/dl)
Diabetes Pregnancy Study Group (DPSG) of the European Association for the Study of Diabetes (EASD) [54]	2 hr 75 g OGTT	Fasting: >5.2 mmol/l (93.6 mg/dl)
		OR
		2 hr: >9.0 mmol/l (162 mg/dl)
International Association of Diabetes and Pregnancy Study Groups (IADPSG) [42]	2 hr 75 g OGTT	At least one of the following must be met:
		Fasting: ≥5.1 mmol/l (92 mg/dl)
		1 hr: ≥10.0 mmol/l (180 mg/dl)
		2 hr: ≥8.5 mmol/l (153 mg/dl)
National Diabetes Data Group (NDDG) (1979) [55]	3 hr 100 g OGTT	At least two of the following must be met:
		Fasting: ≥5.8 mmol/l (105 mg/dl)
		1 hr: ≥10.6 mmol/l (190 mg/dl)
		2 hr: ≥9.2 mmol/l (165 mg/dl)
		3 hr: ≥8.0 mmol/l (145 mg/dl)
World Health Organization (1985) [56]	2 hr 75 g OGTT	Fasting: ≥7.8 mmol/l (140 mg/dl)
		OR
		2 hr: ≥7.8 mmol/l (140 mg/dl)
World Health Organization (1999) [4]	2 hr 75 g OGTT	Fasting: ≥7.0 mmol/l (126 mg/dl)
		OR
		2 hr: ≥7.8 mmol/l (140 mg/dl)
World Health Organization (2013) [20]	2 hr 75 g OGTT	At least one of the following must be met:
		Fasting: 5.1–6.9 mmol/l (92–125 mg/dl)
		1 hr: ≥10.0 mmol/l (180 mg/dl)
		2 hr: 8.5–11.0 mmol/l (153–199 mg/dl)

Not only do different testing methods exist but the availability of GDM screening differs from country to country and even within countries. Although it would be ideal to screen every pregnant woman for GDM it is not always feasible from a cost perspective, particularly in low- or middle-income countries (LMICs). In many LMICs, and some high income countries, women tend to be selected for screening only if they fulfil certain GDM risk-associated criteria [17]. Due to this selective screening process one may expect the true extent of GDM in such countries to remain relatively unknown. Furthermore, prevalence rates may be dependent upon the specificity and sensitivity of the selective screening process in identifying at- risk women.

The effects of urbanisation have not only had a profound impact on developing countries’ economies but also on public health. The transition from rural to urban ways of life is often associated with changes in eating habits, body mass and composition, and reduction in physical activity. The movement towards more Westernised diets involves increased consumption of fats, sugars and refined carbohydrates. As a result, LMICs are experiencing a rapid increase in overweight and obesity as well as non-communicable diseases, such as diabetes, that accompany such conditions [1], [18]. Considering this, the prevalence of GDM should be increasing too. Reported prevalence figures for GDM in two high income countries, the United Kingdom and the United States of America, are 2–3% and 2–10% respectively [17]. A study that assessed GDM in the south of India, a LMIC, reported a far greater prevalence of 13.9% [19]. Gestational diabetes mellitus prevalence estimates for another LMIC, Brazil, are thought to be 7.0–7.6% [17].

Diabetes was essentially unknown in Africa in 1901, yet in 2013 19.8 million people were reportedly living with the condition and this number is predicted to increase to 41.5 million in 2035 equating to a 109% increase [20]. In Africa, the movement from a rural lifestyle to a more industrial urbanised way of life is largely responsible for the evolving problem of chronic diseases, of which diabetes is a major contributor [21].

The explosion in the prevalence of diabetes undoubtedly represents a serious public health burden. In addition, it is more than likely to bring along with it a considerable increase in GDM. However, with regards to GDM in Africa, the situation appears relatively unknown. From a cost perspective, many African countries employ a selective screening approach for GDM and the estimated percentage of pregnant women screened is unclear [17]. In order to suggest policy changes regarding screening for GDM, which will ultimately prevent the effects of GDM on the mother and her offspring and in turn reduce the financial and health burden to a country, it is essential that the extent of the condition is well understood. Therefore, we performed a systematic search to identify research into diagnostic strategies, screening approaches and reported GDM prevalence figures on the African continent.

## Methods

### Protocol and Registration

This project was not prospectively registered. A protocol was developed during the planning process.

### Information Sources and Search Strategy

The PRISMA guidelines (Checklist S1) for the reporting of systematic reviews were followed [22]. Two authors (SM and SAN) independently performed a literature search using three electronic databases; PubMed, Scopus and the Cochrane Library. The following search terms and combinations were used: “gestational diabetes” and Africa; “impaired fasting glucose” and pregnancy and Africa; diabetes and pregnancy and Africa; “impaired glucose tolerance” and pregnancy and Africa; “gestational diabetes” and “African countries.” In addition, the search terms “gestational diabetes,” together with the names of each individual country in Africa were used. For example, “gestational diabetes” and Egypt; “gestational diabetes” and Namibia; “gestational diabetes” and “South Africa” were entered into the search. The list of all 54 recognised African countries included in the search can be found in Appendix S1. Finally, “gestational diabetes” and “Sub-Saharan Africa” were searched for. Where possible, filters were set for studies pertaining to humans but articles written in all languages were included. The search was performed in September 2013. No time limits were set in an attempt to gather all articles published up until the end of September 2013. Once duplicate references were removed the titles and abstracts of the references were screened.

Studies pertaining to African countries that included the following were considered relevant:

1. Screening methods for GDM
2. Criteria used to diagnose GDM
3. Prevalence of GDM

If an article failed to mention any of the above three points it was excluded. In addition, studies were excluded if they were:

1. On type 1 and/or type 2 diabetes only
2. Overviews of GDM
3. Editorials
4. Molecular studies
5. Solely on the outcomes and/or problems associated with macrosomic infants with no reference to GDM prevalence and screening
6. Focussed on perinatal mortality and congenital abnormality rates in babies born to mothers with diabetes
7. Solely comparisons of GDM testing regimes

### Data Extraction

Full text articles were obtained and reviewed. Data were then extracted regarding country, region (rural/urban), population group, sample size, age of pregnant women in the cohort, gestational age, how the investigators defined GDM, how they tested for GDM and what GDM prevalence was reported. In addition, data were also extracted from abstracts that included how GDM was screened for, what criteria were used and what prevalence figures were obtained in the study but for which full text articles could not be obtained.

### Assessment of Reporting Quality and Risk of Bias

The reporting quality of each study was assessed using the Strengthening the Reporting of Observational Studies in Epidemiology (STROBE) checklist [23] guided by the published detailed explanation on how to use the checklist [24]. The combined checklist designed for cohort, case-control and cross-sectional studies was utilised (Appendix S2). A quality assessment score out of 22 was determined for each study by assigning a point per STROBE item addressed. Good/fair quality papers were categorised as having a score of ≥14/22 and poor quality papers were classified as having a score of <14/22. All studies, regardless of their STROBE score, were retained in the systematic review.

Bias was assessed using the Risk of Bias Tool for Prevalence Studies developed by Hoy, Brooks, Woolfe et al., (2012) [25], adapted specifically for this systematic review (Appendix S3). The tool consists of ten items which address four areas of bias and an eleventh item includes a summary risk of bias assessment. The items assess both external and internal validity. Each study was rated as having a low, moderate or high risk of bias. Studies were classified as having a low risk of bias when eight or more of the ten questions were answered as “yes (low risk)”, a moderate risk of bias when six to seven of the questions were answered as “yes (low risk)” and a high risk of bias when five or fewer questions were answered as “yes (low risk)”.

## Results

### Study Selection

**Figure 1:**
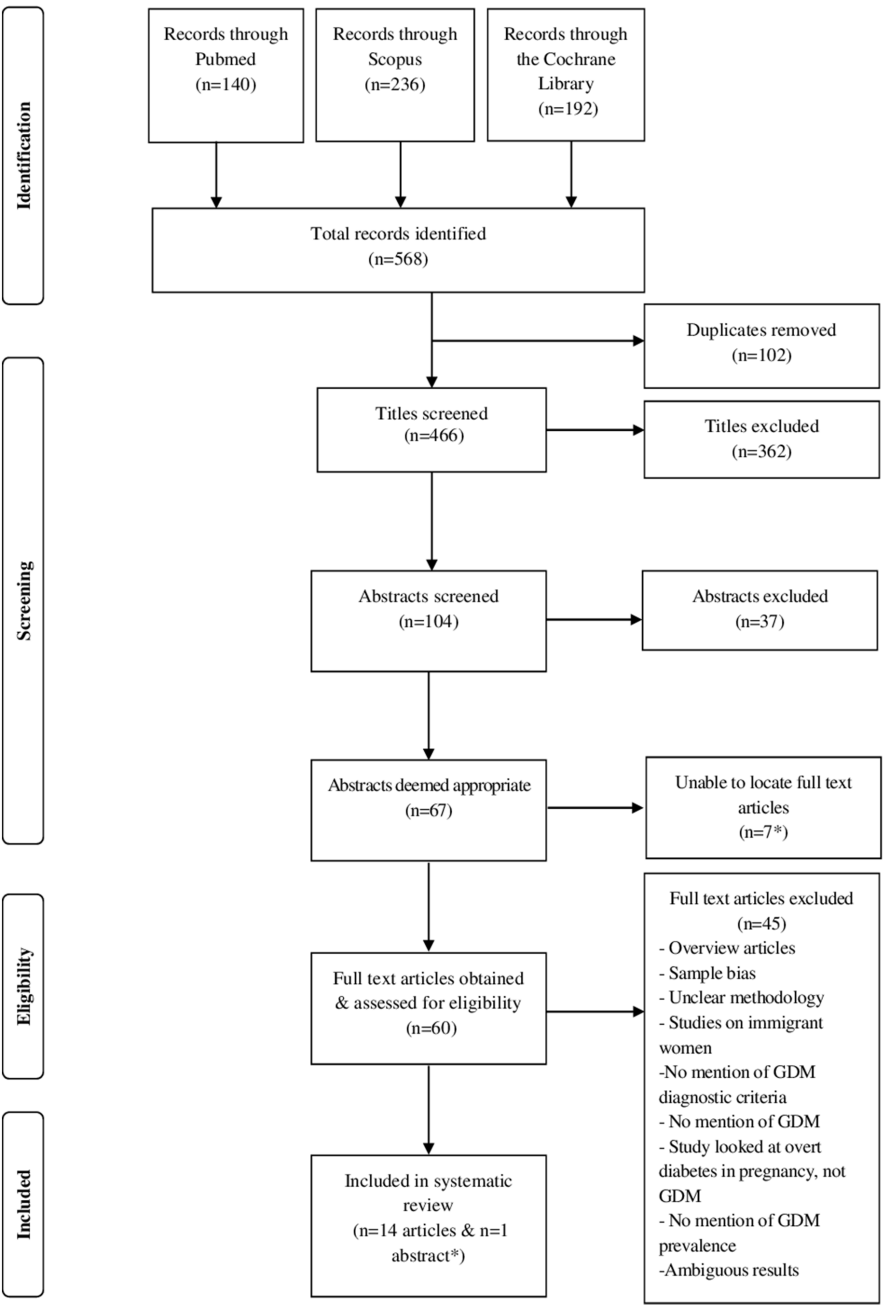
Flow diagram illustrating the number of included and excluded studies in the systematic review on gestational diabetes mellitus in Africa.

The three databases searched identified a total of 568 records. A total of 102 duplicates were removed resulting in 466 unique records after which 362 records were excluded based on their titles being considered irrelevant to the search topic. Of the 104 abstracts screened, 67 abstracts were considered to be relevant. Due to lack of access to the particular journals, despite several attempts, seven full text articles could not be obtained. After reviewing the full text articles of 60 of the records, 14 met all the criteria for the systematic review. In addition, one abstract, for which the full text article could not be obtained, was also considered relevant to the systematic review. A French-speaking colleague read, translated and extracted data from the one article written in French. Articles that were excluded were those in which information regarding classification of, diagnostic criteria for and screening methods for GDM was missing, where methodology was unclear and where investigations were performed on immigrant women as opposed to women representative of the local pregnant population (Figure 1).

### Reporting Quality and Risk of Bias

The STROBE scores per study and the risk of bias results are listed in Table 2. Quality and risk of bias assessments were not performed on the study for which only an abstract could be obtained [26] and for the systematic review that provided details on that one particular study [27]. With regards to reporting quality and referring to the STROBE checklist (Appendix S2), describing the study design, sources of bias, statistical methods used and study limitations were areas where a number of the studies fell short.

**Table 2:** Reporting quality and risk of bias assessments.

Author	STROBE reporting quality score*	Overall risk of bias
Seyoum et al., 1999 [29]	18/22	Low
Bouhsain et al., 2009 [30]	16/22	High
Challis et al., 2002 [31]	11/22	Moderate
Olarinoye et al., 2004 [32]	18/22	Low
Adegbola & Ajayi, 2008 [33]	17/22	Moderate
Kamanu et al., 2009 [34]	19/22	High
Kuti et al., 2012 [27]	19/22	Moderate
Anzaku & Musa, 2013 [35]	17/22	Low
Ozumba et al., 2004 [36]	12/22	High
Jackson & Coetzee, 1979 [37]	15/22	Moderate
Ranchod et al., 1991 [38]	16/22	Low
Mamabolo et al., 2006 [39]	18/22	Moderate
Basu et al., 2010 [40]	19/22	High
Swai et al., 1991# [26]	Not assessed	Not assessed

*Good/fair quality papers were categorised as having a score of ≥14/22, poor quality papers were classified as having a score of <14/22.

# As only the abstract was available an assessment of the reporting quality and risk of bias could not be performed.

Out of the 13 studies that underwent a risk of bias assessment, four (31%) were considered to have a high risk of bias; five were classified as having a moderate risk of bias (38%) and four (31%) were considered to have a low risk of bias.

### Study Characteristics

Thirteen original research articles, one systematic review article and one abstract pertaining to an original research study were finally included in the systematic review thus totalling 14 African research studies (Figure 1). The systematic review article [28] discussed studies in Sub-Saharan Africa and contained suitable information concerning the study for which only an abstract was available. The earliest study was published in 1979 and the latest in 2013, therefore the original individual studies included in the review involved research spanning 35 years. Overall, information regarding GDM classification, screening methods and prevalence was obtained for six African countries; Ethiopia, Morocco, Mozambique, Nigeria, South Africa and Tanzania. Two of the 14 studies looked at GDM prevalence amongst women with risk factors (selective screening), another three studies were case control studies assessing GDM prevalence amongst women at increased risk for the condition versus women without risk factors, and the remaining nine studies involved universal GDM screening of pregnant women. With reference to Table 3:

**Table 3:** Prevalence of Gestational Diabetes Mellitus (GDM) in Africa.

Author	Country	Region(rural/urban)	Populationgroup	Samplesize	Age of women	Gestational age when tested for GDM	GDM diagnosticcriteria used	Diagnostic test used to determine GDM	GDM prevalence
Seyoum et al., 1999 [29]	Ethiopia	Tigray (rural)	Black	890	27.4±7.1yrs(15–50 yrs)	24+ weeks	WHO criteria (1985)*	2 hr 75 g OGTT	3.7% (33/890)
Bouhsain et al., 2009 [30]	Morocco	De Rabat (urban)	Not stated	426	28.8±6.1 yrs	24–28 weeks	Carpenter and Coustan’s criteria*	3 hr 100 g OGTT	7.7% (8/426)
Challis et al.,2002 [31]	Mozam-bique	Maputo (urban/suburban)	Not stated (assumed Black)	Cases: 109(women withlate fetal deaths)	Mean of 25 yrs	>27 weeks	Fasting blood glucose of ≥6.7 mmol/l (120.6 mg/dl) and/or OGTT 2 hr blood glucose of ≥9.0 mmol/l (162 mg/dl)	2 hr 75 g OGTT	11% (12/109 cases)
				Controls: 110(women withlive births)		Post delivery			7.3% (8/110 controls)
Olarinoye et al., 2004 [32]	Nigeria	Lagos (urban)	Black	248 (138; 75 gOGTT, 110; 100 g OGTT)	30.7±4.2 yrs(18–41 yrs)	≥28 weeks	WHO criteria (1985)* −75 g OGTT	3 hr 75 g OGTT	11.6% (16/138) −75 g OGTT
							NDDG criteria (1979)*- 100 g OGTT	3 hr 100 g OGTT	4.5% (5/110)- 100 g OGTT
Adegbola & Ajayi,2008 [33]	Nigeria	Lagos (urban)	Black	Cases: 113women with risk factors	19–45 yrs	24–28 weeks and repeated at 30–32 weeks	Carpenter and Coustan’s criteria*	3 hr 100 g OGTT	6.2% (7/113 cases)
				Controls: 109 womenwithout risk factors					4.6% (5/109 controls)
Kamanu et al., 2009 [34]	Nigeria	Aba (urban)	Black	Cases: 240womenwith macrosomic babies	19–45 yrs	24–28 weeks	1 hr 50 g OGTT >7.8 mmol/l (140 mg/dl).	1 hr 50 g OGTT	2.5% (6/240 cases)
							Borderline results: 2 hr 75 g OGTT plasma glucose level >10 mmol/l (180 mg/dl) at 1 hr and >8.6 mmol/l (154.8 mg/dl) at 2 hr	Borderline cases followed up with a 2 hr 75 g OGTT	
				Controls: 8800 womenwith normalweight babies					1.5% (134/8800 controls)
Kuti et al., 2012 [27]	Nigeria	Ibadan (urban)	Black	765	19–45 yrs	4–40 weeks	WHO criteria (1999)*	2 hr 75 g OGTT	13.9% (106/765) (amongst women with risk factors)
Anzaku & Musa, 2013 [35]	Nigeria	Jos (urban)	Black	253	19–42 yrs	24–28 weeks	WHO criteria (1985)*	2 hr 75 g OGTT	8.3% (21/253)
Ozumba et al., 2004 [36]	Nigeria	Enugu (urban)	Black	12030	15–54 yrs	≥28 weeks	WHO criteria (1999)*	2 hr 75 g OGTT	1% (122/12030)
Jackson & Coetzee, 1979 [37]	South Africa	Cape Town (urban)	Not stated	558	Not stated	All gestations (test repeated in 3^rd^ trimester)	When 2 of the following 3 criteria were exceeded on 2 separate GTT:	2 hr 50 g OGTT	3% (17/558) (amongst women with risk factors)
							2) Maximum level: 10.0 mmol/l (180 mg/dl) (excluding the 30 min figure)		
							3)2 hr level: 6.7 mmol/l(120.6 mg/dl)		
Ranchod et al., 1991 [38]	South Africa	Pieter-martizburg (urban)	Indian (majority) and Coloured (minority)	1717	Not stated	28–32 weeks	WHO criteria (1985)* and DSPG of EASD criteria (1988)*	2 hr 75 g OGTT	3.8% (65/1717): WHO
									1.6% (27/1717): DPSG of EASD
Mamabolo et al., 2006 [39]	South Africa	Limpopo (rural)	Black	262	25.5±6.9 yrs	28–36 weeks	WHO criteria (1999)*	2 hr 75 g OGTT	8.8% (23/262)
Basu et al., 2010 [40]	South Africa	Johannesburg (urban)	Black (94%), White (4%), Mixed (1.7%) and Asian (0.5%)	767	13–31 yrs	23–32 weeks	Institutional protocol: Fasting blood glucose: >8.0 mmol/l (180 mg/dl) or random blood glucose: 11.0 mmol/l (198 mg/dl)	Fasting or random blood glucose levels	1.8% (14/767)
Swai et al., 1991** [26]; Hall et al., 2011[28]	Tanzania	Unknown (rural)	Black	189	Unavailable	Unavailable	WHO criteria (1985)*	2 hr 75 g OGTT	0% (0/189)

*Refer to Table 1;

**Could not obtain full text article.

#### Ethiopia

Only one study on GDM in rural Ethiopia, performed over a decade ago, was included. This was a well reported study with a low risk of bias. The OGTT was utilised as the diagnostic test based on the WHO 1985 criteria and a GDM prevalence of 3.7% was reported [29].

#### Morocco

The one article pertaining to research performed in urban Morocco was published in 2009 and was written in French. The authors reported a relatively high prevalence of GDM; 7.7% using the Carpenter and Coustan’s criteria. However, the authors stated that all women who tested positive on a glucose challenge screening test should have then been referred for an OGTT yet only 40% of these women received an OGTT. This suggests that the GDM prevalence could actually have been higher if all women requiring an OGTT were in fact tested. The authors did report that the GDM prevalence was similar to the prevalence of type 2 diabetes in that population. Unfortunately no reference was made to the ethnicity of the study participants and considering Morocco has several ethnic groups it is difficult to say who this prevalence figure applies to [30]. In addition, the risk of potential bias within this study was high.

#### Mozambique

Only one case control study, of relatively poor reporting quality and moderate risk of bias, was analysed from Mozambique. The study was conducted in 2002 in an urban/suburban setting and the population group was not stated. Considering the majority of the Mozambican population is black, it is assumed that the cohort consisted of black females. Authors of the study reported a GDM prevalence of 11% amongst women who had late fetal deaths (cases) and 7.3% amongst women who had delivered live new-borns (controls). The investigators diagnosed GDM using their own diagnostic criteria which classified glucose readings for diabetes mellitus and IGT as GDM [31].

#### Nigeria

Six Nigerian studies, all on urban populations, were evaluated. These studies were conducted between the years 2004–2013 [27], [32]–[36]. Five of the six studies were classified as having good/fair reporting quality and one was classified as poor. The risk of bias across the six studies ranged between low, moderate and high. All the studies used the OGTT as the method to detect GDM but different glucose concentrations were employed (50 g, 75 g and 100 g) over a time period of one to three hours.

One study focussed solely on determining the prevalence of GDM amongst women with risk factors which included (i) history of fetal macrosomia; (ii) maternal obesity; (iii) previous intrauterine death; (iv) first degree relative with diabetes; (v) glycosuria and (vi) history of GDM in a previous pregnancy [27]. Another two studies were case control studies whereby women with risk factors for GDM [33] or women who had delivered macrosomic babies [34] were classified as cases, and women without risk factors [33] or women who had delivered normal weight babies [34] served as the controls. Prevalence of GDM was higher amongst the cases in both studies; 6.2% versus 4.6% (utilising the Carpenter and Coustan’s criteria) [33] and 2.5% versus 1.5% (utilising the investigators own diagnostic criteria) [34]. However, Kamanu et al., (2009), who used their own diagnostic criteria as mentioned above, diagnosed GDM based on a 1 hour 50 g OGTT (>7.8 mmol/l/140 mg/dl) and only followed up borderline results with a 75 g 2 hour OGTT [34]. Usually the 50 g glucose load is referred to as a glucose challenge test and women who test positive on the challenge test are followed up with a further OGTT. This is referred to as the two step approach [5]. It is unconventional for a 50 g OGTT to be performed independently as a diagnostic test and so the results of this study could be questionable.

Excluding the two case-control studies discussed above, the other four Nigerian studies utilised the WHO diagnostic criteria (two used the WHO 1985 criteria and two used the WHO 1999 criteria). One of these four studies compared the detection rate of the three hour 75 g OGTT using the WHO 1985 criteria to the three hour 100 g OGTT using the NDDG criteria. The 75 g OGTT with WHO 1985 diagnostic criteria yielded a higher GDM prevalence (11.6% versus 4.5%). Conversely, this study found that the incidence of fetal macrosomia was higher (66.7%) amongst women diagnosed with GDM by the 100 g OGTT using the NDDG criteria than amongst women diagnosed with GDM by the 75 g OGTT using the WHO 1985 criteria (23.1%) [32].

#### South africa

Four South African studies, conducted between 1979 and 2010, were included in the systematic review [37]–[40]. One study focused predominantly on Indian women [38], two on black women [39], [40] and the other did not state the ethnicity of the women [37]. The study by Jackson and Coetzee (1979) tested women for GDM because they had one or more risk factors. These risk factors included (i) a parent or sibling with diabetes; (ii) repeated miscarriages; (iii) obesity; (iv) previous macrosomic infant; (v) glycosuria; (vi) previous hyperglycaemia; (vii) previous infant with a severe congenital anomaly; (viii) previous perinatal death; (ix) polyhydramnios and (x) Indian ethnicity. In addition, this particular study utilised a 2 hour 50 g OGTT and the investigators’ own diagnostic criteria [37]. A 50 g glucose load is usually used for the glucose challenge test and an OGTT generally utilises either 75 g or 100 g of glucose [5]. The glucose load chosen for an OGTT by the investigators is unusual. However, this study was performed in 1979 and can therefore be considered outdated. Optimisation of the OGTT for the diagnosis of GDM has developed and improved greatly since then.

All but one study employed a two hour OGTT for the diagnosis of GDM. The one study that did not employ an OGTT was interestingly the most recent study in South Africa, conducted in 2010, which tested fasting or random blood glucose levels and referred to an institutional protocol for diagnostic criteria [40]. Ranchod et al., (1991) compared the WHO 1999 criteria and DSPG of EASD criteria; WHO criteria produced a higher GDM prevalence (3.8% versus 1.6%) [38]. Overall, the four South African studies produced GDM prevalence figures ranging from 1.6% to 8.8%.

#### Tanzania

One study, published in 1991, was included on GDM prevalence in rural Tanzania [26]. Unfortunately, the full text article could not be obtained but data was extracted from the abstract and the review article [28]. This study involved an OGTT on a small sample of women (n = 189) using the WHO 1985 diagnostic criteria. A prevalence of 0% was determined. Unfortunately, as the full text article could not be obtain, reporting quality and risk of bias for this study could not be assessed.

## Discussion

As far as the authors are aware, no other systematic review has assessed the prevalence of GDM across the African continent. This systematic review therefore focussed on studies in African countries that provided details on the GDM screening methods employed, the diagnostic criteria used and the prevalence figures obtained.

Africa consists of 54 countries [41] yet only six African countries, equating to a mere 11%, were represented in this systematic review. The percentage of countries for which prevalence figures were found in a systematic review that assessed GDM in Asia was 26% [42]. Although still low, this regional representation is better than the one found in the current review. This highlights the fact that little seems to be known about the prevalence and potential burden of GDM in African countries. Before health care policies and guidelines can successfully be drawn up and implemented, it is important for one to establish the extent of a particular problem. It is evident that the extent of GDM in Africa as a whole is not well investigated. Africa has been plagued with under-nutrition and GDM may not be considered a public health concern. However, as African countries shift economically a double burden of under- and over-nutrition emerges. With the increase in over-nutrition, particularly in females, GDM may be naively overlooked.

The results of the systematic review illustrate that the majority of the studies tested for GDM at around 24–28 weeks gestation, the recommended gestational age for when an OGTT should be performed [42]. In addition, the most commonly employed method for GDM screening in Africa is the two hour 75 g OGTT with glucose reference ranges as stipulated by the WHO 1985 or 1999 diagnostic criteria (Table 3). Two of the reported studies made comparisons between different diagnostic criteria and screening methods. One of the Nigerian studies showed that the two hour 75 g OGTT using the WHO 1985 criteria diagnosed more than double the amount of women that the 100 g OGTT using the NDDG criteria [32]. In addition, one of the South African studies also illustrated a two-fold detection rate using the 1985 WHO criteria versus the DSPG of EASD criteria [38]. Based on these findings, whether the 75 g OGTT over-diagnoses GDM in women is debatable and warrants further investigation. This statement is supported by the authors of the systematic review on GDM Asia who commented that the choice of diagnostic criteria greatly affects GDM prevalence [43].

Many lessons have been learnt from the Hyperglycemia and Adverse Pregnancy Outcomes (HAPO) study which showed that there is a continuous association between maternal blood glucose levels below those diagnostic of diabetes, and adverse outcomes, such as increased neonatal birth weight [44]. As a result of these findings various groups have reconsidered the diagnostic criteria for GDM. The IADSPG diagnostic criteria and WHO 2013 diagnostic criteria are not as stringent as some of the other/previous criteria mainly because only one abnormal value, as opposed to two, is sufficient to make a diagnosis of GDM (Table 1). As a result of using the newer criteria it is very likely that the prevalence of GDM will increase. This has both positive and negative consequences. For example, more women will be diagnosed with GDM and receive treatment and management which in turn will decrease the effects of maternal hyperglycaemia on the mother and developing fetus. On the other hand, the health system in a country could become overburdened with GDM pregnancies, which could impact heavily on a country’s economy [45]. However, considering the potential adverse pregnancy outcomes and the long term effects of GDM on mother and baby, it may be beneficial to the individuals, as well as a country’s health system and economy, to diagnose and manage more women than less. None of the studies reported in this systematic review used the WHO 2013 or IADPSG criteria.

The percentage of women affected with GDM in this review was as low as 0% in rural Tanzania [26] and as high as 13.9% amongst urban Nigerian women with risk factors [27]. This disparity in prevalence is possibly due to the different methodology and study designs employed across the 14 studies. Without the availability of a standardised universal screening protocol the question is raised as to whether or not the prevalence figures that were obtained through the various studies are in fact true reflections of the African situation. In addition, with respect to the discussion above regarding the newer IADSPG and WHO 2013 diagnostic criteria, should the 14 studies reported in this systematic review have utilised either of the said criteria the GDM prevalence figures obtained would most likely have been greater.

Two of the studies, one performed in Nigeria and the other in South Africa, only tested women with risk factors for GDM and therefore employed the selective screening approach within their methodology [27], [37]. Certain risk factors have indeed been proven to be very useful in identifying women at risk for GDM; when BMI is >30 versus <20 kg/m^2^ a woman has a three times greater risk of developing GDM. Ethnicity is also another key factor for assessing the risk of developing GDM; Asian women are five times more likely to develop GDM than Caucasian women, and African-American women are two times more likely to develop GDM than Caucasian women [2]. The study by Kuti et al., (2012) in Nigeria reported a high GDM prevalence (13.9%) amongst these women and the authors found the strongest associations between the following risk factors and a diagnosis of GDM: being over 30 years of age (although this was not used as a risk factor in the sample selection process), having a family history of diabetes and having previously been diagnosed with GDM [27].

The South African study that tested women with risk factors produced a much lower prevalence of GDM (3%) but did report a strong association between glycosuria, previous hyperglycaemia and having two or more of the listed risk factors with a diagnosis of GDM [37]. These studies support that certain maternal risk factors have a high specificity in identifying women at risk of developing GDM. This selective screening approach may certainly have an important role in resource-limited settings.

The countries with the most studies pertaining to GDM were South Africa and Nigeria, which had four and six studies reported respectively. With particular reference to South Africa, considering there are 22 million black females living in the country, representing approximately 80% of the entire female population [44], two studies on GDM in black women, one in a rural setting [39] and one in an urban setting [40], involving a total cohort of approximately 983 women, cannot be considered representative of the South African GDM scenario. In addition, out of the six African countries for which GDM prevalence figures were obtained, only Nigeria and South Africa have reported relatively recent figures on macrosomia rates. In Nigeria it is thought that macrosomia accounts for 7.5% [45] to 8.1% [46], [47] of births which ties in with the high GDM prevalence figures of 8.3% [35] and 13.9% [27] as reported by the two Nigerian studies in this review. This suggests macrosomia may be a marker for GDM prevalence. With respect to South Africa, one study conducted on black patients in urban Soweto reported a 2.3% macrosomia prevalence [48] but recent unpublished data from the South African Department of Health indicates a surprisingly low macrosomia rate of 1.7% [49]. If macrosomia rates are indicative of GDM rates then it is imperative that research on GDM is conducted in other African countries. Algeria and Uganda’s macrosomia prevalence figures are reported as 14.9% and 8.4% respectively [45], this raises concern regarding their possible GDM figures.

It is alarming that very little appears to be known about GDM in African countries. Research studies, such as those listed in this systematic review, and particularly those that screen all women in the study cohort for GDM, are exceptionally useful in assessing the prevalence of the problem. Based on the 14 reported studies included in the systematic review, if one ignores the prevalence figures obtained from the two studies that focussed on higher risk women [27], [37] and takes the prevalence of GDM amongst the control group in the case control studies [31], [33], [34], and selects the prevalence figures obtained by the WHO diagnostic criteria as opposed to those obtained by the NDDG criteria in one study [32] and the DSPG of EASD criteria in another study [38], the overall prevalence of GDM in Africa is estimated to be approximately 5% (60.1/12); approximately two and a half to seventeen times greater than some high income countries (Denmark (2–3%), the UK (2–3%) Germany (0.3–0.8%)) [17].

Interestingly, few studies were performed on rural populations. As a direct consequence of urbanisation it would be expected that the prevalence of GDM would be higher amongst urban populations as opposed to rural populations. Out of the four South African studies (three urban and one rural) the study in rural Limpopo produced the highest GDM prevalence (8.8%) amongst a representative sample of local pregnant women [39]. However, one of the limitations in making comparisons between the rural and urban studies in this review is the different GDM screening methods employed and diagnostic criteria used. In addition, some studies looked at women already at high risk for GDM. Other limitations to this review include only published studies, as opposed to grey literature, being searched and roughly one third of the studies included in the review having a high risk of bias and another third having a moderate risk of bias.

This systematic review has illustrated a gap in the knowledge of GDM in Africa with only 11% of the African continent being represented. More epidemiological based studies on GDM in African countries need to be performed in order to provide reliable information and thus clarity on the extent of GDM. An ideal scenario would be if one set of diagnostic criteria and one testing method was employed across the continent in order to produce comparable data. In addition, comparisons between GDM prevalence amongst rural and urban populations within a country should be carried out in order to assess the extent of the effects of urbanisation on public health.

Understanding and subsequently attempting to curb the prevalence of GDM in developing countries is imperative for maternal and child health. As GDM often results in macrosomic infants, birth trauma and the need for Caesarean sections at delivery are expected. This is precarious as it impacts both maternal and child survival during delivery, and places a significant economic burden on the health system, which in many African countries is already struggling with limited resources.

Furthermore, for most countries macrosomia appears to have been overlooked with the justified focus on low birth weight and small for gestational age statistics. The Developmental Origins of Health and Disease research describes how the developing fetus is susceptible to its environment and that certain *in utero* events can in fact alter fetal programming and produce different phenotypes. Low birth weight is representative of poor fetal nutrition and growth, and has been shown to be associated with a range of chronic conditions, including type 2 diabetes [50]. However, high birth weight requires as much consideration as there is evidence to support that fetal over-nutrition also poses risk for type 2 diabetes and other chronic conditions later in life [51]. With the emerging increase in type 2 diabetes and obesity, macrosomia will become an important factor in maternal and child health and should be reported on and monitored by the health care system as a marker for GDM sooner than later.

As Africa continues along its economic and concomitant urbanisation and lifestyle transitions, the double burden of both under- and over-nutrition is a cause for concern. Therefore, epidemiologists, public health specialists, health professionals, and policy leaders need to place GDM and macrosomia as key elements in their maternal and child health framework, thus enabling policies and practice to minimise the risk of maternal impaired glucose metabolism during pregnancy.

## Supporting Information

Checklist S1 **PRISMA 2009 checklist.**
(DOC)

Appendix S1 **The 54 countries in Africa according to the United Nations.**
(DOCX)

Appendix S2 **STROBE Statement: checklist of items that should be included in reports of observational studies.**
(DOC)

Appendix S3 **Risk of bias assessment tool.**
(DOCX)
