# Supplementary material for: Gestational Diabetes Mellitus in Africa: A Systematic Review
Source: PLoS One. 2014 Jun 3;9(6):e97871. doi: 10.1371/journal.pone.0097871 (PMC4043667; doi:10.1371/journal.pone.0097871)
Supplement: Appendix S1 — The 54 countries in Africa according to the United Nations. (DOCX) [file pone.0097871.s002.docx]

**Appendix S1 The 54 countries in Africa according to the United Nations**

(Taken from: http://www.worldatlas.com)

1. Algeria
2. Angola
3. Benin
4. Botswana
5. Burkina
6. Burundi
7. Cameroon
8. Cape Verde
9. Central African Republic
10. Chad
11. Comoros
12. Congo
13. Congo, Democratic Republic of
14. Djibouti
15. Egypt
16. Equatorial Guinea
17. Eritrea
18. Ethiopia
19. Gabon
20. Gambia
21. Ghana
22. Guinea
23. Guinea-Bissau
24. Ivory Coast
25. Kenya
26. Lesotho
27. Liberia
28. Libya
29. Madagascar
30. Malawi
31. Mali
32. Mauritania
33. Mauritius
34. Morocco
35. Mozambique
36. Namibia
37. Niger
38. Nigeria
39. Rwanda
40. Sao Tome and Principe
41. Senegal
42. Seychelles
43. Sierra Leone
44. Somalia
45. South Africa
46. South Sudan
47. Sudan
48. Swaziland
49. Tanzania
50. Togo
51. Tunisia
52. Uganda
53. Zambia
54. Zimbabwe
