# Supplementary material for: Gestational Diabetes Mellitus in Africa: A Systematic Review
Source: PLoS One. 2014 Jun 3;9(6):e97871. doi: 10.1371/journal.pone.0097871 (PMC4043667; doi:10.1371/journal.pone.0097871)
Supplement: Appendix S3 — Risk of bias assessment tool. (DOCX) [file pone.0097871.s004.docx]

**Appendix S3 Risk of bias assessment tool**

Adapted from the Risk of Bias Tool for Prevalence Studies developed by Hoy, Brooks, Woolfe et al. (2012)

| **Risk of Bias Item** | **Answer:**  **Yes (Low Risk) or No (High risk)** |
| --- | --- |
|  |  |
| **External Validity** |  |
| 1. Was the study target population a close representation of the national pregnant population in relation to relevant variables? |  |
| 1. Was the sampling frame a true or close representation of the target population? |  |
| 1. Was some form of random selection used to select the sample, OR, was a census undertaken? |  |
| 1. Was the likelihood of non-participation bias minimal? |  |
| **Internal Validity** |  |
| 1. Were data collected directly from the subjects? (as opposed to medical records) |  |
| 1. Were acceptable diagnostic criteria for GDM used? |  |
| 1. Was a reliable and accepted method of testing for GDM utilised? |  |
| 1. Was the same mode of data collection used for all subjects? |  |
| 1. Was GDM tested for within the advised gestational period of 24-28 weeks? |  |
| 1. Were the numerator(s) and denominator(s) for the calculation of the prevalence of GDM appropriate? |  |
| 1. Summary item on the overall risk of study bias   LOW RISK OF BIAS: 8 or more “yes” answers. Further research is very unlikely to change our confidence in the estimate.  MODERATE RISK OF BIAS: 6 to 7 “yes” answers. Further research is likely to have an important impact on our confidence in the estimate and may  change the estimate.  HIGH RISK OF BIAS: 5 or fewer “yes” answers. Further research is very likely to have an important impact on our confidence in the estimate and is likely  to change the estimate. |  |
